# Supplementary material for: Intestinal microbiome composition and its relation to joint pain and inflammation
Source: Nat Commun. 2019 Oct 25;10:4881. doi: 10.1038/s41467-019-12873-4 (PMC6814863; doi:10.1038/s41467-019-12873-4)
Supplement: Supplementary file 1 — Supplementary Information [file 41467_2019_12873_MOESM1_ESM.pdf]

Intestinal microbiome composition and its relation to joint pain and inflammation

Boer *et al.*

Supplementary Information:

Supplementary Figures:

Supplementary Figure 1: PCA plot of Aitchison distances of inter-individual microbial composition in the Rotterdam Study - Page 2

Supplementary figure 2: Spearman correlation between 16s sequencing and qPCR *Streptococcus* relative abundancies in the Rotterdam Study – Page 3

Supplementary Tables:

Supplementary Table 1: Association results of α-diversity metrics and β-diversity with Knee OA pain and severity in the Rotterdam Study – Page 4

Supplementary Table 2: streptococcus spp. association with WOMAC-knee pain score in Rotterdam Study is independent of alcohol use, smoking and BMI – Page 5

Supplementary Table 3: Streptococcus spp. association with WOMAC-knee pain score in Rotterdam Study. CEU ancesty only- Page 6

Supplementary Table 4: Streptococcus spp. association with WOMAC-knee pain score in Rotterdam Study is not driven by oral medication use – Page 7

Supplementary Table 5: Increased Streptococcus spp. associated with increased WOMAC-knee-pain- Page 8

Supplementary Table 6: Streptococcus spp. Association with WOMAC-pain score when using ILR and ARS transformation – Page 9

Supplementary Table 7: General characteristics of the replication cohort Lifelines Deep – Page 10

Supplementary Table 8: Increased relative abundancy of Streptococcus is associated with increased Knee WOMAC-pain score, when adjusting for BMI – Page 11

Supplementary Table 9: List of all Primers used in this study – Page 12

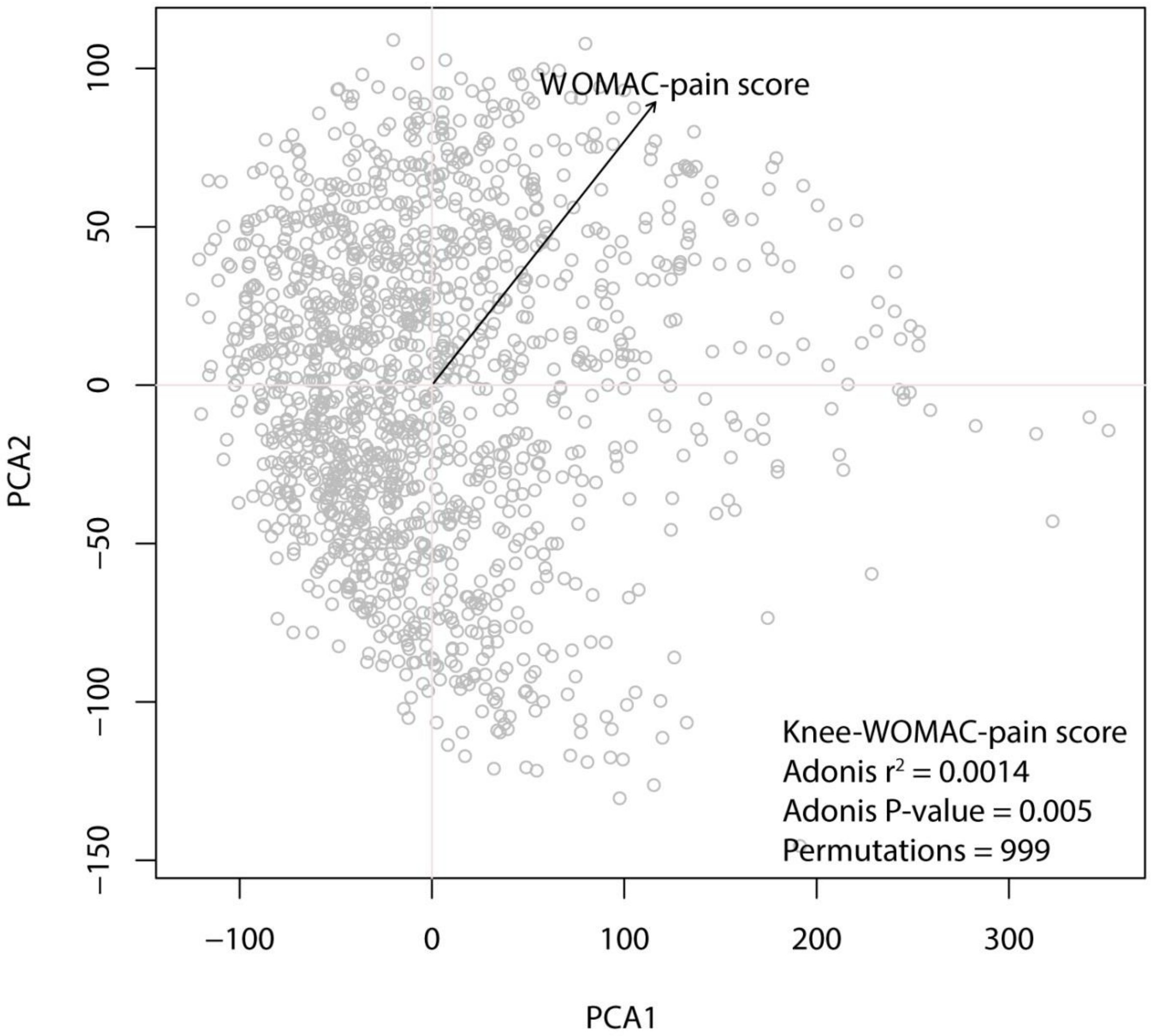

Supplementary Figure 1: PCA plot of Aitchison s distances of inter-individual microbial composition in the Rotterdam Study. With plotted vector of knee WOMAC-pain score. PERMANOVA P-value calculated through VEGAN R package ADONIS function, permutations=999, n=1,427

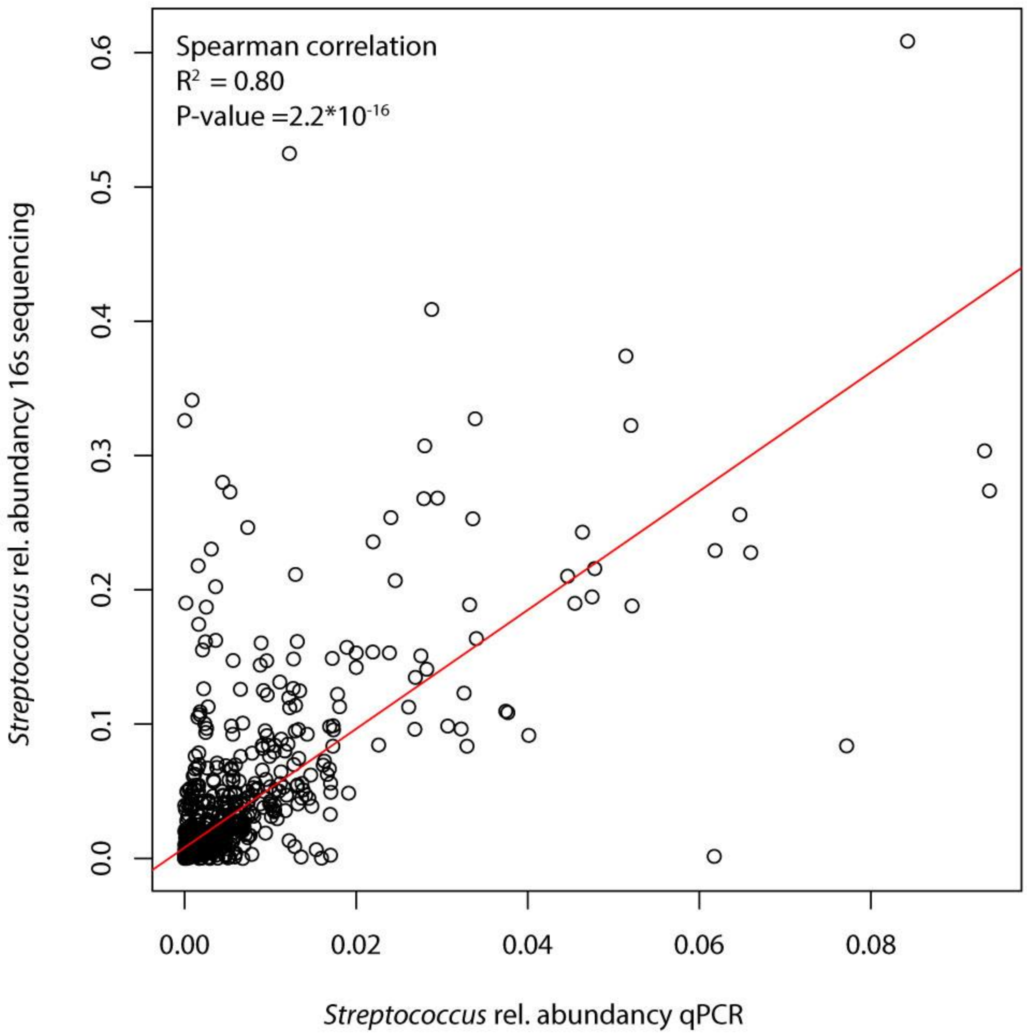

Supplementary Figure 2: Spearman correlation between 16s sequencing and qPCR *Streptococcus* relative abundancies in the Rotterdam Study**.** Spearman correlation between relative *Streptococcus spp.* abundancy determined by 16S rRNA sequencing and quantitative *Streptococcus spp.* abundancy determined by qPCR, in the Rotterdam study microbiome. Red line depicts the regression line (16S sequencing abundancy ~ qPCR abundancy), N=1,427

Supplementary Table 1: Association results of α-diversity metrics and β-diversity with Knee OA pain and severity in the Rotterdam Study

	Knee KLsum		Knee WOMAC pain	
	**Beta / r^2**	**P-Value**	**Beta / r^2**	**P-value**
**Shannon index**	$-9.5*10^{-02}$	0.31	$-2.3*10^{-01}$	0.07
**Inverse Simpson index**	$-1.6*10^{-03}$	0.69	$-8.9*10^{-03}$	0.13
**B-diversity**	$1.39*10^{-03}$	0.09	$1.4*10^{-03}$	$5.0*10^{-03}$

α-diversity metrics used are the Shannon Index, Inverse Simpson Index. Knee KLSum is the sum of the Kellgren-Lawrence score in the left knee and right knee. WOMAC-pain score is a sum of the WOMAC pain questionnaire question for the left and right knee. Poisson regression model was used. For Beta-diversity we calculated the Aitchison distance from CLR normalized data, P-values and r2 were calculated using adonis PERMANOVA for 999 iterations. All analysis used were adjusted for adjusted for age, sex, TimeInMail and Batch

S**upplementary Table 2**: Streptococcus spp. association with WOMAC-knee pain score in Rotterdam Study is independent of alcohol use, smoking and BMI

		**Model 1**			**Model 2**			**Model 3**		
		**Age + Sex + Technical CoVar.**			**Model 1 + Smoking + Alcohol**			**Model 2 + BMI**		
**Taxonomy**	**N**	**CoE**	**P-Value**	**FDR**	**CoE**	**P-Value**	**FDR**	**CoE**	**P-Vale**	**FDR**
**Class .| *Bacilli***	1,419	$6.07*10^{-03}$	$9.06*10^{-09}$	$2.32*10^{-06}$	$5.81*10^{-03}$	$3.19*10^{-08}$	$8.16*10^{-06}$	$5.00*10^{-03}$	$2.44*10^{-06}$	$6.3*10^{-04}$
**Order ..| *Lactobacillales***	1,417	6.11*10-^{03}	$7.61*10^{-09}$	$2.32*10^{-06}$	$5.85*10^{-03}$	$2.65*10^{-08}$	$8.16*10^{-06}$	$5.03*10^{-03}$	$2.20*10^{-06}$	$6.3*10^{-04}$
**Family … | *Streptococcaceae***	1,402	4.91*10-^{03}	$1.52*10^{-07}$	$1.94*10^{-05}$	$4.70*10^{-03}$	$4.52*10^{-07}$	$5.78*10^{-05}$	$4.01*10^{-03}$	$2.10*10^{-05}$	$2.7*10^{-03}$
**Genus …. | *Streptococcus***	1,396	5.04*10-^{03}	$7.28*10^{-08}$	$1.24*10^{-05}$	$4.83*10^{-03}$	$2.20*10^{-07}$	$3.76*10^{-05}$	$4.13*10^{-03}$	$1.22*10^{-05}$	$2.1*10^{-03}$

MaAslin results for Streptococcus WOMAC-pain score association. FDR is based on full dataset (256 taxonomies). Model 1: the base model of WOMAC + age + sex + technical covariates (Technical CoVar: Time in mail + Batch). Model 2 : the base model with Alcohol use and smoking added. Model 3: model 2 with BMI added. T.Covar: technical Covariates, CoE: Coefficient, FDR: Benjamin-Hochberg FDR multiple testing corrected P-value, BMI: Body Mass Index. Analysis done on full dataset (256 taxonomies). P-value, FDR and CoE from MaAslin analysis.

Supplementary Table 3: Streptococcus spp. association with WOMAC-knee pain score in Rotterdam Study. CEU ancesty only.

		Model 1 Age + Sex + Technical CoVar.			Model 2 Model 1 + Smoking + Alcohol			Model 3 Model 2 + BMI		
**Taxonomy**	**N**	**CoE**	**P-Value**	**FDR**	**CoE**	**P-Value**	**FDR**	**CoE**	**P-Vale**	**FDR**
**Class .| *Bacilli***	1,257	$5.83*10^{-03}$	$7.58*10^{-08}$	$9.70*10^{-06}$	$5.67*10^{-03}$	$1.54*10^{-07}$	$1.98*10^{-05}$	$5.11*10^{-03}$	$3.06*10^{-06}$	$3.92*10^{-04}$
**Order ..| *Lactobacillales***	1,255	$5.87*10^{-03}$	$6.46*10^{-08}$	$9.70*10^{-06}$	$5.72*10^{-03}$	$1.30*10^{-07}$	$1.98*10^{-05}$	$5.14*10^{-03}$	$2.77*10^{-06}$	$3.92*10^{-04}$
**Family … | *Streptococcaceae***	1,243	$5.06*10^{-03}$	$2.98*10^{-07}$	$2.54*10^{-05}$	$4.94*10^{-03}$	$5.65*10^{-07}$	$4.82*10^{-05}$	$4.43*10^{-03}$	$9.39*10^{-06}$	$8.02*10^{-04}$
**Genus …. | *Streptococcus***	1,237	$4.99*10^{-03}$	$9.05*10^{-07}$	$5.79*10^{-05}$	$4.86*10^{-03}$	$1.66*10^{-06}$	$1.06*10^{-04}$	$4.30*10^{-03}$	$2.91*10^{-05}$	$1.86*10^{-03}$

MaAslin results for Streptococcus WOMAC-pain score association on individuals with CEU ancestry only. CEU (central european ancestry) was determined by genetic principal components. Individuals with a minimum of 50% CEU ancestry were included. FDR is based on full dataset (256 taxonomies). Model 1: the base model of WOMAC + age + sex + technical covariates (Technical CoVar: Time in mail + Batch). Model 2 : the base model with Alcohol use and smoking added. Model 3: model 2 with BMI added. T.Covar: technical Covariates, CoE: Coefficient, FDR: Benjamin-Hochberg FDR multiple testing corrected P-value, based on full dataset (256 taxonomies), BMI: Body Mass Index. P-value, FDR, CoE from MaAslin analysis.

Supplementary Table 4: Streptococcus spp. association with WOMAC-knee pain score in Rotterdam Study is not driven by oral medication use

		Model 3		Model 3		Model 3		Model 3		
		+ NSAID use		+ PPI use		+ NSAID use + PPI use		+ Exclusion of PPI users		
**Taxonomy**	**N**	**CoE**	**P-value**	**CoE**	**P-value**	**CoE**	**P-value**	**N**	**CoE**	**P-value**
**Class .| *Bacilli***	1,419	$5.09*10^{-03}$	$2.04*10^{-06}$	$4.20*10^{-03}$	$4.06*10^{-05}$	$4.16*10^{-03}$	$5.25*10^{-05}$	1,123	$3.25*10^{-03}$	$7.96*10^{-04}$
**Order ..| *Lactobacillales***	1,417	$5.11*10^{-03}$	$1.90*10^{-06}$	$4.22*10^{-03}$	$3.76*10^{-05}$	$4.18*10^{-03}$	$4.89*10^{-05}$	1,121	$3.31*10^{-03}$	$6.29*10^{-04}$
**Family … | *Streptococcaceae***	1,402	$4.06*10^{-03}$	$1.98*10^{-05}$	$3.32*10^{-03}$	$2.65*10^{-04}$	$3.27*10^{-03}$	$3.61*10^{-04}$	1,109	$2.38*10^{-03}$	$3.67*10^{-03}$
**Genus …. | *Streptococcus***	1,396	$4.17*10^{-03}$	$1.22*10^{-05}$	$3.43*10^{-03}$	$1.66*10^{-04}$	$3.37*10^{-03}$	$2.38*10^{-04}$	1,104	$2.40*10^{-03}$	$3.97*10^{-03}$

MaAslin results for Streptococcus WOMAC-pain score association. Model 3: base model of WOMAC + age + sex + technical covariates with smoking, alcohol consumption and BMI added. T.Covar: technical Covariates, CoE: Coefficient, BMI: Body Mass Index. NSAID: non-steroidal anti-inflammatory drugs, and PPI: Proton Pump Inhibitors. N: nr of individuals in the analysis. P-value and CoE from MaAslin analysis.

Supplementary Table 5: Increased Streptococcus spp. associated with increased WOMAC-knee-pain score is not driven by WOMAC distribution or outliers

<table>
<tr><th></th><th colspan="5">WOMAC-Pain (>0)</th><th colspan="5">WOMAC-Pain (<10)</th></tr>
<tr><th></th><th></th><th colspan="2">Model 1</th><th colspan="2">Model 3</th><th></th><th colspan="2">Model 1</th><th colspan="2">Model 3</th></tr>
<tr><th>Taxonomy</th><th>N</th><th>CoE</th><th>P-value</th><th>CoE</th><th>P-value</th><th>N</th><th>CoE</th><th>P-value</th><th>CoE</th><th>P-value</th></tr>
<tr><td>Class .| Bacilli</td><td>313</td><td>$5.34*10^{-03}$</td><td>$9.90*10^{-03}$</td><td>$4.29*10^{-03}$</td><td>$3.97*10^{-02}$</td><td>1,392</td><td>$5.67*10^{-03}$</td><td>$2.00*10^{-05}$</td><td>$4.49*10^{-03}$</td><td>$7.40*10^{-04}$</td></tr>
<tr><td>Order ..| Lactobacillales</td><td>312</td><td>$5.34*10^{-03}$</td><td>$1.01*10^{-02}$</td><td>$4.27*10^{-03}$</td><td>$4.10*10^{-02}$</td><td>1,390</td><td>$5.70*10^{-03}$</td><td>$1.88*10^{-05}$</td><td>$4.50*10^{-03}$</td><td>$7.29*10^{-04}$</td></tr>
<tr><td>Family … | Streptococcaceae</td><td>309</td><td>$6.06*10^{-03}$</td><td>$7.51*10^{-04}$</td><td>$5.40*10^{-03}$</td><td>$3.14*10^{-03}$</td><td>1,375</td><td>$4.39*10^{-03}$</td><td>$3.13*10^{-04}$</td><td>$3.41*10^{-03}$</td><td>$5.31*10^{-03}$</td></tr>
<tr><td>Genus …. | Streptococcus</td><td>306</td><td>$6.25*10^{-03}$</td><td>$5.29*10^{-04}$</td><td>$5.56*10^{-03}$</td><td>$2.38*10^{-03}$</td><td>1,369</td><td>$4.53*10^{-03}$</td><td>$2.03*10^{-04}$</td><td>$3.53*10^{-03}$</td><td>$3.93*10^{-03}$</td></tr>
</table>

Model 1: WOMAC score knee adjusted for age, sex and technical covariates. Model 3: model 1 adjusted for smoking, alcohol use and BMI. CoE: Coefficient, FDR: Benjamin-Hochberg FDR multiple testing corrected P-value, based on full analysis (248 taxonomies) in MaAslin, N = nr of individuals included in the analysis

Supplementary table 6: Streptococcus spp. Association with WOMAC-pain score when using ILR and ARS transformation

			Model 1		Model 2		Model 3	
			Age + Sex + Technical CoVar		Model 1 + Alcohol + Smoking + BMI		Model 3 + NSAIDs + PPI	
**Taxonomy**	**Method**	**N**	**CoE**	**P-value**	**CoE**	**P-value**	**CoE**	**P-value**
**Genus …. | *Streptococcus***	ARS	1,395	$5.04*10^{-03}$	$7.28*10^{-08}$	$4.13*10^{-03}$	$1.22*10^{-05}$	$2.40*10^{-03}$	$3.97*10^{-03}$
**Genus …. | *Streptococcus***	ILR	1,395	$9.28*10^{-02}$	$9.87*10^{-06}$	$7.01*10^{-02}$	$8.35*10^{-04}$	$5.38*10^{-02}$	$8.49*10^{-03}$

ARS: arcsince square root transformation applied in MaAslin analysis, ILR: isometric log ratio transformation, ILR was done on full genus level taxonomy datset. CoE: coefficient, Technical CoVar: technical covariates consiting of TimeInMail and Batch. P-value and CoE, from MaAslin analysis.

Supplementary Table 7: General characteristics of the replication cohort Lifelines Deep

General Characteristics	Females	Males	Total
**Cohort participants**	505	362	867
**Age (years)**	45.6(12.9)	45.7(13.2)	45.6(13)
**BMI (Kg/m2)**	25.1(4.6)	25.4(3.4)	25.2(4.2)
**Alcohol (gram/day)**	6.2(6.6)	10.8(11.1)	8.1(9)
**Smoking (y/n)**	87 smokers/416 non-smokers	58 smokers/303 non-smokers	145 current smokers
**PPI (y/n)**	42 users/444 non-users	22 users/326 non-users	64 current PPI users
**NSAIDs (y/n)**	17 users/469 non-users	11 users/337 non-users	28 current NSAID users
**Knee Phenotypes**			
**Knee OA (y/n)**	x	x	x
**KLSum score**	x	x	x
**WOMAC-Pain score**	1.2(2.8)	0.5(1.7)	0.9(2.4)
**WOMAC-Pain score > 0**	133	64	197
**α-diversity metrics**			
**Shannon Index**	3.8(0.4)	3.7(0.3)	3.7(0.4)
**Inverse Simpson Index**	22.2(8)	20.9(7.2)	21.7(7.7)

PPI: Proton Pump Inhibitor, BMI: Body Mass Index, NSAID: non-steroidal anti-inflammatory drugs, Klsum score: Kellgren-Lawrence sum score of the knee joints

Supplementary Table 8. Increased relative abundancy of Streptococcus is associated with increased Knee WOMAC-pain score, when adjusting for BMI

			Rotterdam Study			Life Lines Deep				Meta-Analysis	
**Taxonomy**	**%**	**N**	**CoE**	**SE**	**P-Value**	**N**	**CoE**	**SE**	**P-value**	**N**	**P-Vale**
**Class . | *Bacilli***	27.3%	1,419	$5.17*10^{-03}$	$1.06*10^{-03}$	$1.21*10^{-06}$	867	$5.14*10^{-03}$	$1.30*10^{-03}$	$8.47*10^{-05}$	2,286	$3.5*10^{-10}$
**Order .. | *Lactobacillales***	100%	1,417	$5.20*10^{-03}$	$1.06*10^{-03}$	$1.10*10^{-06}$	864	$4.53*10^{-03}$	$1.33*10^{-03}$	$6.82*10^{-04}$	2,281	$2.6*10^{-09}$
**Family … | *Streptococcaceae***	79.6%	1,402	$4.16*10^{-03}$	$9.43*10^{-04}$	$1.12*10^{-05}$	863	$2.72*10^{-03}$	$1.28*10^{-03}$	$3.47*10^{-02}$	2,265	$1.5*10^{-06}$
**Genus …. | *Streptococcus***	98.7%	1,396	$4.28*10^{-03}$	$9.45*10^{-04}$	$6.41*10^{-06}$	860	$3.03*10^{-03}$	$1.59*10^{-03}$	$5.74*10^{-02}$	2,256	$1.1*10^{-06}$

MaAslin analysis results, adjusted for age, sex, technical covariates: DNA isolation batch and time In mail, and BMI. Rotterdam Study (n=1,427), Life Lines Deep (n=867), Meta-analysis: Rotterdam Study and Life Lines Deep together, weighted meta-analysis in METAL. Taxonomy% = percentage of taxonomy is from one taxonomy level higher, ex. 23.7% of all Firmicutes are Bacilli. N = number of individuals in cohort where microbial abundancy is not zero for that taxonomy. FDR: P-value adjusted for multiple testing, Benjamin-Hochberg False Discovery Rate, CoE: Coefficient, SE: Standard Error.

Supplementary Table 9: List of all primers used in this study

Primer	Sequence	Target	Ref
**319F**	ACTCCTACGGGAGGCAGCAG	V3 region	[1]
**515F**	GTGCCAGCMGCCGCGGTAA	V4 region	[2]
**806R**	GGACTACHVGGGTWTCTAAT	V4 region	[1,2]
**U16SRTF**	ACTCCTACGGGAGGCAGCAGT	16S	[3]
**U16SRTR**	TATTACCGCGGCTGCTGGC	16S	[3]

V3/V4 regions: hypervariable region 3,4 of the bacterial 16S rRNA gene, 16S: bacterial 16S rRNA gene.

References:

1. Fadrosh, D. W. et al. An improved dual-indexing approach for multiplexed 16S rRNA gene sequencing on the Illumina MiSeq platform. Microbiome 2, 6 (2014).
2. Fu, J. et al. The Gut Microbiome Contributes to a Substantial Proportion of the Variation in Blood Lipids. Circ. Res. 117, 817–824 (2015).
3. Clifford, R. J. et al. Detection of Bacterial 16S rRNA and Identification of Four Clinically Important Bacteria by Real-Time PCR. PLoS One 7, e48558 (2012).
