# Supplementary material for: Intestinal microbiome composition and its relation to joint pain and inflammation
Source: Nat Commun. 2019 Oct 25;10:4881. doi: 10.1038/s41467-019-12873-4 (PMC6814863; doi:10.1038/s41467-019-12873-4)
Supplement: Supplementary file 3 — Description of Additional Supplementary Files [file 41467_2019_12873_MOESM3_ESM.docx]

**Description of Additional Supplementary Files**

**File Name: Supplementary Data 1**

**Description:** Rotterdam Study gastrointestinal microbiome association with WOMAC-knee pain score, adjusted for age, TimeInMail and batch. Total nr of tested taxonomies was 596, after MaAslin automatic QC 256 taxonomies remained.
