# Supplementary material for: Intestinal microbiome composition and its relation to joint pain and inflammation
Source: Nat Commun. 2019 Oct 25;10:4881. doi: 10.1038/s41467-019-12873-4 (PMC6814863; doi:10.1038/s41467-019-12873-4)
Supplement: Supplementary file 5 — Reporting Summary [file 41467_2019_12873_MOESM5_ESM.pdf]

nature research

Corresponding author(s): Joyce B. J. van Meurs (j.vanmeurs@erasmusmc.nl)

Last updated by author(s): Aug 20, 2019

Reporting Summary

Nature Research wishes to improve the reproducibility of the work that we publish. This form provides structure for consistency and transparency in reporting. For further information on Nature Research policies, see Authors & Referees and the Editorial Policy Checklist .

Statistics

For all statistical analyses, confirm that the following items are present in the figure legend, table legend, main text, or Methods section.

n/a	Confirmed	
☐	☒	The exact sample size (n) for each experimental group/condition, given as a discrete number and unit of measurement
☐	☒	A statement on whether measurements were taken from distinct samples or whether the same sample was measured repeatedly
☐	☒	The statistical test(s) used AND whether they are one- or two-sided<br>*Only common tests should be described solely by name; describe more complex techniques in the Methods section.*
☐	☒	A description of all covariates tested
☐	☒	A description of any assumptions or corrections, such as tests of normality and adjustment for multiple comparisons
☐	☒	A full description of the statistical parameters including central tendency (e.g. means) or other basic estimates (e.g. regression coefficient) AND variation (e.g. standard deviation) or associated estimates of uncertainty (e.g. confidence intervals)
☐	☒	For null hypothesis testing, the test statistic (e.g. F, t, r) with confidence intervals, effect sizes, degrees of freedom and P value noted<br>*Give P values as exact values whenever suitable.*
☒	☐	For Bayesian analysis, information on the choice of priors and Markov chain Monte Carlo settings
☒	☐	For hierarchical and complex designs, identification of the appropriate level for tests and full reporting of outcomes
☐	☒	Estimates of effect sizes (e.g. Cohen's d, Pearson's r), indicating how they were calculated

Our web collection on statistics for biologists contains articles on many of the points above.

Software and code

Policy information about availability of computer code

Data collection	Software used was an in house pipeline utilizing QIIME version 1.90 and UPARSE version 8.1 for sequencing read analysis and quality control. we used RDP classifier 2.12 with SILVA 16S rRNA database (128) for taxonomic calling of the sequencing reads
Data analysis	All statistical analysis were performed in R: A Language and Environment for Statistical Computing. We have used the following R packages for analysis: VEGAN (Permanova and ordination),MaAsLin (multivariate linear regression) and CoDaSeq (ILR and CLR).

For manuscripts utilizing custom algorithms or software that are central to the research but not yet described in published literature, software must be made available to editors/reviewers. We strongly encourage code deposition in a community repository (e.g. GitHub). See the Nature Research guidelines for submitting code & software for further information.

Data

Policy information about availability of data

All manuscripts must include a data availability statement. This statement should provide the following information, where applicable:

- Accession codes, unique identifiers, or web links for publicly available datasets
- A list of figures that have associated raw data
- A description of any restrictions on data availability

The Source data for figure 1 and Supplementary Figures 1 and 2 are provided with the paper. The data supporting the findings of this study are not allowed to be published in open or closed data repositories due to the General Data Protection Regulation (GDPR). However, the data can be made available upon reasonable request. For requests regarding the Rotterdam Study data and Rotterdam Study microbiome dataset please contact Frank van Rooij (f.vanrooij@erasmusmc.nl). . All other correspondence and material requests should be addressed to J.B.J.v.M (j.vanmeurs@erasmusmc.nl).

Field-specific reporting

Please select the one below that is the best fit for your research. If you are not sure, read the appropriate sections before making your selection.

- [x] Life sciences
- [ ] Behavioural & social sciences
- [ ] Ecological, evolutionary & environmental sciences

For a reference copy of the document with all sections, see nature.com/documents/nr-reporting-summary-flat.pdf

Life sciences study design

All studies must disclose on these points even when the disclosure is negative.

Sample size	No sample sizes were calculated, Same size was selected based on availability of the data, i.e., amount of participants in the Rotterdam Study (RS-III-2) of whom microbiome and WOMAC-pain measurements were taken.
Data exclusions	Participants were excluded if they had used antibiotics in the 6 month prior to stool sample collection, stool samples took longer than 3 days to reach the Erasmus MC or if the 16S rRNA sequencing read count was <10,000 reads for that participant.
Replication	16S rRNA measurements were validated using qPCR and association results were replicated in an independent cohort (n=867)
Randomization	Not relevant: we have an independent cohort for replication.
Blinding	There was no group allocation in our study. Individuals who have done the sample collection and sample analysis did not perform the statistical analysis.

Reporting for specific materials, systems and methods

We require information from authors about some types of materials, experimental systems and methods used in many studies. Here, indicate whether each material, system or method listed is relevant to your study. If you are not sure if a list item applies to your research, read the appropriate section before selecting a response.

Materials & experimental systems

n/a	Involved in the study	
x		Antibodies
x		Eukaryotic cell lines
x		Palaeontology
x		Animals and other organisms
	x	Human research participants
x		Clinical data

Methods

n/a	Involved in the study	
x		ChIP-seq
x		Flow cytometry
x		MRI-based neuroimaging

Human research participants

Policy information about studies involving human research participants

Population characteristics	Rotterdam Study (RS), cohort III. visit 2 microbiome set (=n=14,27, 821 females, 606 males). chohort characteristics= avrage (standard deviation: age (years)=56.9 (5.9), BMI (kg/m2)=27.5 (4.5), Alcohol(glass/day)=1.3 (2.6), WOMAC-pain=0.9 (2.3), Smoking (y/n)=195 current smokers
Recruitment	RS-III: recruitment of individuals aged >45, living in the Ommoord district, and not already included in RS-i or RS-II.
Ethics oversight	Medical Ethical Committee of Erasmus MC (University Medical Center Rotterdam, The Netherlands, MEC 02.1015)

Note that full information on the approval of the study protocol must also be provided in the manuscript.
